# Challenge in the Pathological Diagnosis of the Follicular-Patterned Thyroid Lesions

**DOI:** 10.31557/APJCP.2021.22.10.3365

**Published:** 2021-10

**Authors:** Dalia A Elsers, Mahmoud R A Hussein, Mohammed Hassan Osman, Ghada A Mohamed, Ghada Hosny

**Affiliations:** 1 *Faculty of Medicine, Assiut University, Egypt. *; 2 *Department of Pathology Faculty of Medicine, Assiut University, Assiut, Egypt. *; 3 *Department of Head and Neck Surgery, Faculty of Medicine, Assiut University, Assiut, Egypt. *; 4 *Department of Internal Medicine Faculty of Medicine, Assiut University, Assiut, Egypt. *

**Keywords:** FPTLs, NIFTP, HBME-1, CK19, Galectin 3, CD56

## Abstract

**Background:**

The follicular-patterned thyroid lesions (FPTLs) include hyperplastic nodules (HN), follicular adenoma (FA), non-invasive follicular neoplasm with papillary-like nuclear features (NIFTP), follicular carcinoma (FC), and the follicular variant of papillary carcinoma (FVPTC). Sometimes the pathologists cannot accurately separate these lesions from each others on a histological basis.

**Aims:**

To evaluate the utility of immunohistochemistry in the diagnosis of FPTLs.

**Materials and methods:**

Immunohistochemical analysis, incorporating 83 cases of histologically confirmed FPTLs out of which 20 carcinomas, 51 benign FPTLs (38 HN and 13 FA), and 12NIFTP were separated from each others using four immunostains (HBME-1, CK19, Galectin-3, and CD56). Results: We found statistically significantly more frequent expression of HBME-1, CK19, Galectin-3 proteins in carcinomas as compared to benign FPTLs (p = <0.01). HBME-1 and Galectin-3 were the most sensitive markers for the diagnosis of malignant FPTLs (75%). Galectin-3 was the most specific marker for the diagnosis of carcinoma (90.3%).

**Conclusions:**

The histomorphological features remain the cornerstone of the diagnosis of FPTN. Although HBME-1, Galectin-3, and CK19 immunostains have some diagnostic value in the separation of malignant from benign FPTLs, they are variably expressed in the benign and malignant FPTLs. No single immunostain has sufficient sensitivity and specificity and therefore their diagnostic use is controversial. Future studies are mandated to find more reliable markers that can separate between benign and malignant FPTLs.

## Introduction

In thyroid pathology, the term “follicular” is used to label lesions of follicular cell origin. It indicates the pattern of growth that builds thyroid follicles with central lumens containing colloid materials. Histologically, a thyroid proliferation is described as follicular when it is entirely or almost entirely (more than 95%) exhibit follicular growth pattern (Baloch and Livolsi, 2002; Baloch and LiVolsi, 2007; Deandrea et al., 2010; Papale et al., 2013). Follicular patterned thyroid lesions (FPTLs) are common, and up to 4 to 7% of the general population develops clinically palpable thyroid nodules (Kasper et al., 1987; Hegedus, 2004). Benign FPTLs are usually thinly-encapsulated or non-capsulated whereas the neoplastic ones are usually capsulated (Baloch and Livolsi, 2002; Baloch and LiVolsi, 2007). FPTLs include several entities such as hyperplastic nodules (HNs), follicular adenoma (FA), non-invasive follicular neoplasm with papillary-like nuclear features (NIFTP), follicular tumors of uncertain malignant potential (FTUMP), follicular carcinoma (FC), follicular variant of medullary carcinoma, and the follicular variant of papillary carcinoma (FVPTC)(Kasper et al., 1987; Baloch and Livolsi, 2002; Baloch and LiVolsi, 2007; Deandrea et al., 2010).

The HNs are usually thinly encapsulated, well-defined masses composed of benign follicles, with occasional degenerative changes or papillary hyperplasia. The non-neoplastic follicular cells usually have round nuclei and can show nuclear grooves but no intranuclear inclusions (Fernandez et al., 1997; Abd El Atti and Shash, 2012).The FAs are usually solitary thinly encapsulated mass, composed of follicular cells arranged in follicles, cords, sheets, and rarely papillae. The thorough sampling of FAs reveals lack of nuclear features of PTC, lymphovascular or capsular invasion (Nucera et al., 2005; Wang et al., 2019). The non-invasive FVPTC has recently been reclassified as NIFTP. These premalignant lesions are usually well-defined, encapsulated, composed of follicles with no papillary structures, capsular or vascular invasion. The constituent cells show PTC nuclear features but no hemorrhage or necrosis. The FVPTC may be encapsulated or non-encapsulated. It is composed almost entirely of neoplastic follicles, with nuclear features of PTC (Kasper et al., 1987; Baloch and Livolsi, 2002; Baloch and LiVolsi, 2007; Deandrea et al., 2010). 

Sometimes the separation of well-differentiated thyroid carcinomas with follicular patterns (FVPTC and FC) from benign FPTLs (HNs, and FAs) is challenging on a histological basis. Therefore, gaining insights into the differences in the expression pattern of some immunostains in FPTLs may help resolve this issue (Hofman et al., 2009) 

These immunostains include HBME-1(named after the laboratory of Dr. Hector Battifora and MEsothelioma), Galectin-3, cytokeratin 19 (CK19), and CD56. The expression of HBME-1, Galectin-3, and CK19 is absent in the normal thyroid follicular cells (Barroeta et al., 2006; Nasr et al., 2006; Chiu et al., 2010; Palanisamy et al., 2017; Abu-Sinna et al., 2018; Rossi and Fadda, 2018; Javalgi et al., 2019). HBME-1 is a marker of mesothelial cells (Donna et al., 1997) that is found in their microvilli (Casey et al., 2003; Nasr et al., 2006; Abu-Sinna et al., 2018; Javalgi et al., 2019). Galectin-3 is a protein of the lectin family which binds to cell surface glycoprotein. It is involved in cellular signaling, cell-cell adhesion, cell-matrix interaction, metastasis, and apoptosis. Galectin-3 is absent in the normal thyroid follicular cells (Barroeta et al., 2006; Chiu et al., 2010; Rossi and Fadda, 2018). CK19 is a small cytokeratin that is involved in organizing myofibers inside the simple and complex epithelial cells (Palanisamy et al., 2017). CD56 is a neural cell adhesion molecule encoded by a gene located at chromosome11q23. It regulates homophilic interactions between neurons. Reduced or loss of CD56 expression is correlated with thyroid cancer (Dunđerović et al., 2015). 

Some previous studies suggested a diagnostic utility of HBME-1, Galectin-3, and CK19, and CD56, in separating malignant PTC from benign FPTLs (such as FA, and HNs), but none of these immunostains is individually reliable to establish firm diagnosis(Dunđerović et al., 2015; Tastekin et al., 2019; Zargari and Mokhtari, 2019). Their main limitation in diagnosis of malignant FPTLs is their inconsistent expression in some benign FPTLs(Niedziela et al., 2002; Mehrotra et al., 2004; Mills et al., 2005; Barroeta et al., 2006; Park et al., 2007; Zhu et al., 2010).Nechifor-Boila, et al examined the expression of HBME-1, Galectin-3, Cytokeratin-19, and CD56 in a series of PTC and FTUMP (Nechifor-Boila et al., 2014). CD56, whose expression is reduced or absent in thyroid carcinomas, was the most sensitive marker (81.8%), showing a “malignant” profile in most PTC. It was followed by HBME-1 (63.6% sensitivity). Cytokeratin-19 and Galectin-3 were the least sensitive antibodies (45.6%), but the most specific ones (100%). In FTUMP, Cytokeratin-19, Galectin-3, HBME-1, and CD56 stained negatively in most of the cases (Nechifor-Boila et al., 2014).

While there have been some studies on individual marker’s expression especially in PTC, we have limited knowledge about the expression profile of all four markers in several benign FPTLs (HNs and FA) and malignant FPTLs (FVPTC, and FC). An understanding of the expression patterns in these lesions will provide valuable insights into the diagnosis of these lesions. To fill this existing gap in the literature, we used immunoperoxidase staining methods to examine the expression patterns of these proteins in the normal thyroid follicular cells, HNs, FA, NIFTP, FC, and FVPTC. 

## Materials and Methods

The Institutional Research Board (IRB) of our institute ethical committee approved this research (0006563 NO: 185/2014)

Study design: The present study was carried out at the Surgical Pathology Laboratory, Faculty of Medicine, Assiut University Hospitals, Assiut University, Assiut, Egypt. The Institutional Ethics and Research Committee approved the design of the investigation. The participant provided written informed consents to use their surgical specimens for scientific research. The study included 83 cases of histologically documented FPTLs (HNs: 38, FA: 13, NIFTP: 12, FVPTC: 26, and FC: 3 cases). All cases were formed entirely (more than 95% of the lesion) of thyroid follicles. Cases of conventional PTC were excluded. The medical records (clinical details), and paraffin blocks were identified and obtained from the tissue archives of our Hospitals. The five-micrometer sections were prepared and stained using routine Hematoxylin and Eosin stains. Histological evaluation of the cases was done by three pathologists (Professor Mahmoud R. Hussein, Dr. Dalia A. EL Sers, and Dr. Ghada Hosny). The pathologists were unaware of the patient clinical data or previously rendered histological diagnosis. In difficult and challenging cases, the diagnosis was made by consensus of two pathologists. The final diagnosis was established based on the recent WHO classification (Lloyd et al., 2017), and the nuclear scoring system proposed by Thompsom et al. (Thompson et al., 2018). 

Immunohistochemical staining procedure: The tissue sections were prepared for immunostaining following other groups (Hussein, 2009). The avidin-Biotin immunoperoxidase staining techniques were performed on three-micrometer tissue sections cut from the most representative formalin-fixed paraffin-embedded tissue blocks. The antigen retrieval methods and antibodies used in this study are summarized in Table 1.Heat-induced epitope antigen retrieval technique was used for 30 minutes at 95°C followed by a 20-minute cool down. The endogenous peroxidase activity was blocked with 10% hydrogen peroxide. Primary antibodies (HBME-1, CK19, Galectin-3, and CD56, Genemed Biotechnologies, Inc, South San Francisco, USA) were applied to each section and incubated at 1 hour for HMBE-1 and CK19 and overnight for Galectin-3 and CD56.Then sections were treated with biotinylated secondary antibodies for 10 min at room temperature following the manufacturer’s instructions (Dako LsAB2 system peroxidase Catalogue K0673,Carpinteria, USA). Proteins (immunocomplex) were finally visualized by exposure to a liquid diaminobenzidine substrate kit. The immunostained sections were counterstained in Mayer’s Hematoxylin stain. All slides were coded and evaluated by three observers blinded for clinical details and the identity of the patients.

Positive and negative controls: Sections with phosphate buffer solution replacing the primary antibody were used as negative controls (Rashad et al., 2011). Positive controls included mesothelioma tissue for HBME-1 (Riera et al., 1997), Ureteric tissue for CK19 (Kasper et al., 1987), papillary thyroid carcinoma for Galectin-3 (Konstantinov et al., 1996), and Small cell lung carcinoma for CD56 antibodies (Tsang et al., 1996). 

Immunohistochemical evaluation: Evaluation of the immunostained sections was done following other groups (Huang et al., 2018). Briefly, the entire section was examined to detect the site and distribution of the staining. HBME-1, Galectin-3, CK19, and CD56 protein expression signals were regarded as positive when immunoreactivity was evident in the cell membrane and/or cytoplasm on light microscopy (magnification, x400). A summary of the staining patterns is shown in Table 1. Initially, the controls were examined and evaluation of the cases was done at the same time. We scored the lack of staining or weak staining in <10% of the lesional cells as negative. The staining in ≥10% of the lesional cells was considered positive. Scoring of the immunohistochemical staining was done based on the extent of staining as negative, focally positive (less than 25% of cells are positive), positive (more than 25% of cells are positive)(Harb, 2017)

*Statistical analysis*

The data were collected, tabulated, and statistically analyzed, using a ‘’Statistical Package for the Social Sciences (SPSS), version 16, (SPSS Inc., Chicago, Illinois, USA) for windows. Descriptive statistics were performed and the results were presented as the mean ± standard deviation. The immunohistochemistry results were compared between the different types of lesions with a Chi-square test (*χ*^2^). Differences were considered as statistically significant when (p-value is ≤ 0.05).

## Results

The clinicopathologic features of FPTLs: This study included 83 cases of FPTLs. The benign FPTLs included 38 HN (10 males and 28 females, with their ages ranging from 18 to 50 years) and 13 FA (4 males and 9 females, with their ages ranging from 20 to 46 years). The mean age of the patient in the benign FPTLs was 35.5. The patients presented clinically with diffuse goiter (15 cases) or solitary nodules (21 cases) or multiple nodules (15 cases). The patients were treated either by lobectomy (18 cases) or subtotal thyroidectomy (33 cases). Grossly, some lesions were thinly encapsulated (10 HN, and 13 FA) whereas others with non-encapsulated (28 HN. A summary of these findings is shown in Table 2. 

We included 12 cases of NIFTP (3 males and 9 females, with their ages ranging from 27 to 45 years, mean age 36.7). The patients presented clinically with solitary nodule (12 cases). The patients were treated either by lobectomy (8 cases) or subtotal thyroidectomy (4 cases). Grossly, all lesions were thinly encapsulated. A summary of these findings is shown in Table 2. 

The malignant FPTLs group included 17 FVPTC (5 males and 12 females, with their ages ranging from 25 to 46 years, mean age 41.5) and 3 cases of FC (1 male and 2 females, with their ages ranging from 40 to 50 years with mean age 45 years ). The mean age of the patients in the malignant FPTLs was 37.7. The patients presented clinically with solitary mass. The patients were treated subtotal thyroidectomy). Grossly, some lesions were thinly encapsulated (10 cases) whereas others with non-encapsulated (7 cases). A summary of these findings is shown in Table 2. 

**Figure 1:**
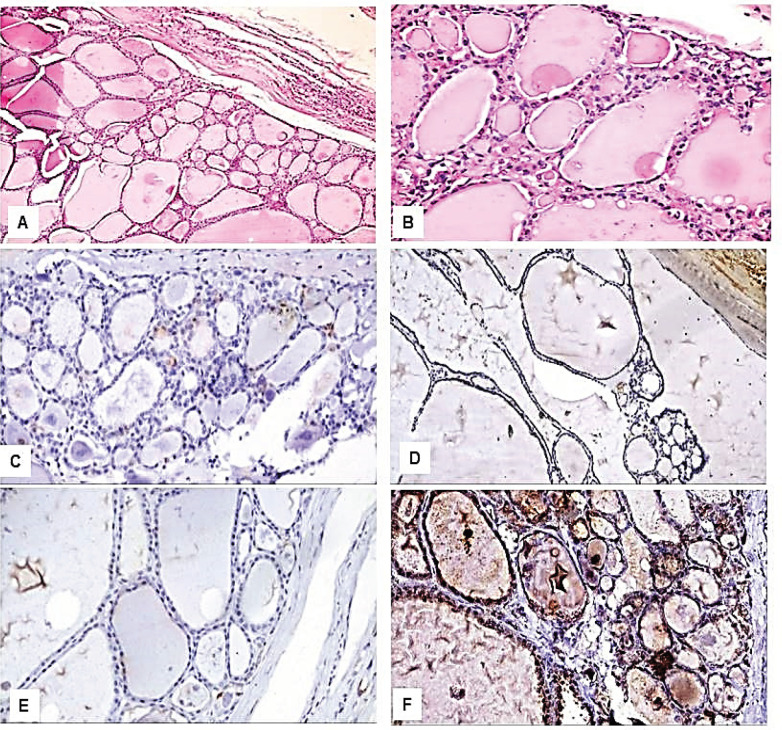
Immunohistological Features of the Hyperplasitc Nodules. A: H&E, x200; B: H&E, x400; C: Negative immunostaining for HBME-1, x400; D: Negative immunostaining for CK19, x400; E: Negative immunostaining for Galactin-3, x400; F: Positive immunostaining for CD56, x400

**Figure 2:**
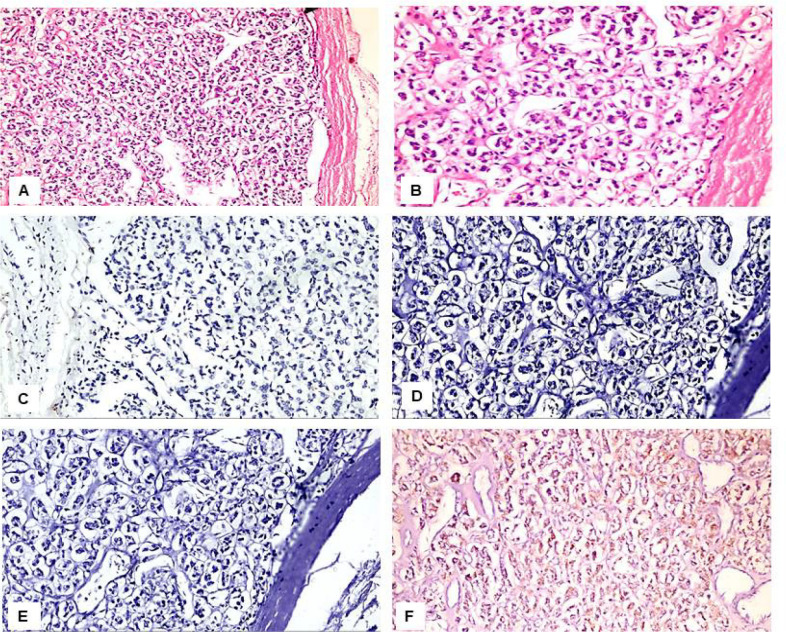
Immunohistological Features of the Follicular Adenoma. A: H&E, x200; B: H&E, x400; C: Negative immunostaining for HBME-1, x400; D: Negative immunostaining for CK19, x400; E: Negative immunostaining for Galactin-3, x400; F: Positive immunostaining for CD56, x400

**Figure 3:**
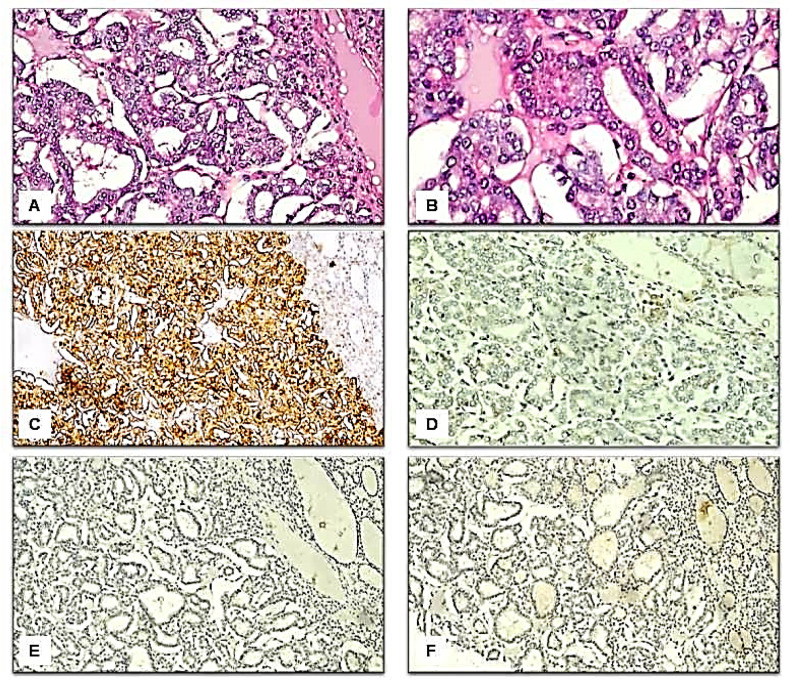
Immunohistological Features of the Non-Invasive Follicular Neoplasm with Papillary-Like Nuclear Features. A: H&E, x200; B: H&E, x400; C: Positive immunostaining for HBME-1, x200; D: Negative immunostaining for CK19, x200; E: Negative immunostaining for Galactin-3, x200; F: Negative immunostaining for CD56, x200

**Figure 4:**
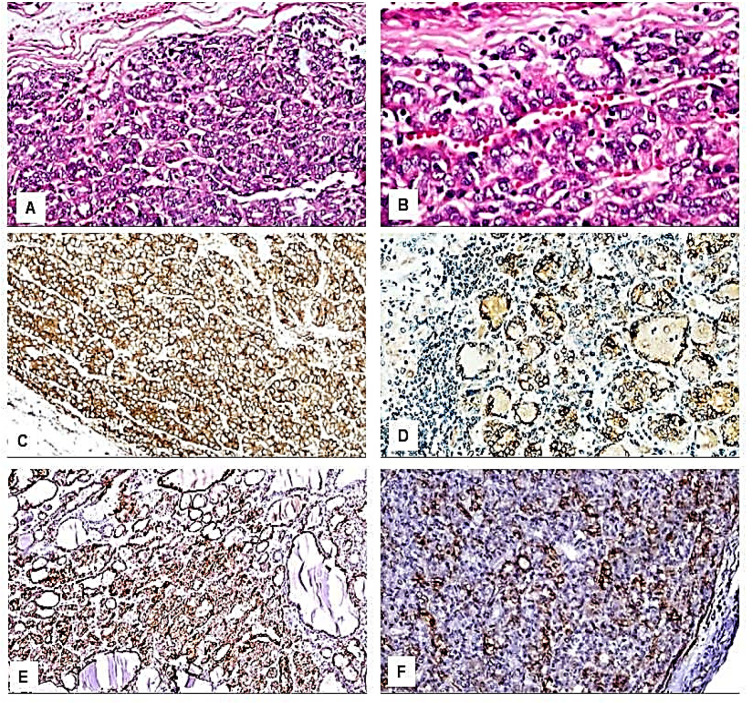
Immunohistological Features of the Follicular Variant of Papillary Thyroid Carcinoma. A: H&E, x200; B: H&E, x400; C: Positive immunostaining for HBME-1, x200; D: Positive immunostaining for CK19, x200; E: Positive immunostaining for Galactin-3, x200; F: Positive immunostaining for CD56, x200

**Figure 5:**
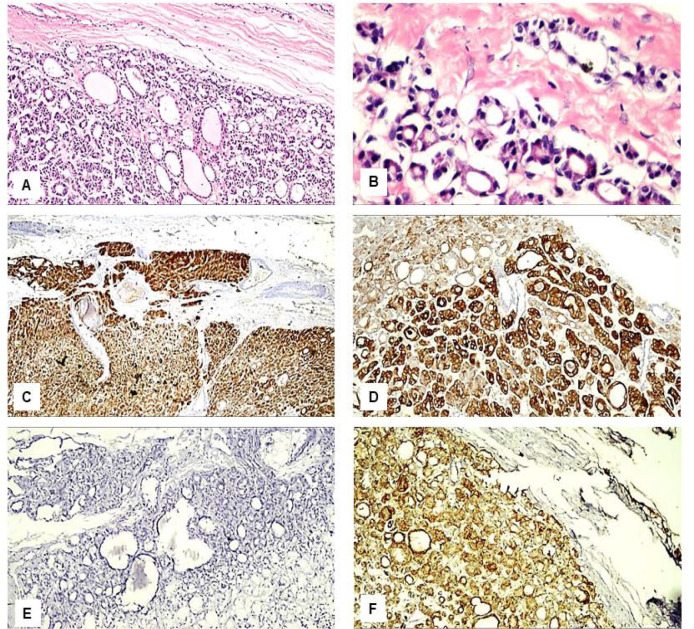
Immunohistological Features of the Follicular Carcinoma. A: H&E, x200; B: H&E, x400; C: Positive immunostaining for HBME-1, x200; D: Positive immunostaining for CK19, x200; E: Negative immunostaining for Galactin-3, x200; F: Positive immunostaining for CD56, x200

Immunohistochemical results: We found variations in the frequency of expression of HBME-1, Galectin-3, CK19, and CD56 proteins between the benign (figure 1 and figure 2), NIFTP (figure 3) and malignant FPTLs (figure 4 and 5). We found significant variations in the expression profile among benign and malignant FPTLs. The frequency of expression of HBME-1, Galectin-3, and CK19 markers was significantly higher in FVPC and FC than in HNs, FA and NIFTP (p = 0.000; p = 0.00, and p=0.02, respectively). A summary of these findings is shown in Tables 3 and 4. 

HBME-1 protein immunoreactivity was not observed in the normal thyroid follicular epithelium. HBME-1 protein expression was observed in 10/51 of benign lesions (15.8% of HNs and 30.8% of FA), in 7/12 (58.3%) NIFTP, and 15/20 carcinomas (70.6% of FVPC and 100% FC). HBME-1expression was absent in 41 cases of benign FPTLs (84.2% of HNs and 69.2% of FA), in 5/12 NIFTP, and5/17 FVPC (29.4%). A summary of these findings is shown in Tables 3 and 4. Galectin-3 protein immunoreactivity was not observed in the normal thyroid follicular epithelium.

Galectin-3 protein expression in the cytoplasm and nucleus was mainly seen in the malignant FPTLs (FVPTC).It was focally seen in 3/51 cases of benign lesions (7.9% of HNs and 0.0 % of FA), in 3/12 (25 %) NIFTP, and in 15/20 carcinomas (52.9% FVPTC and 0.0% of FC). Galectin-3 was absent in 35/38 cases of benign lesions (92.1% of HNs and 100% FA), in 9/12 (75%) NIFTP, in 2/17 FVPTC, and all of the FC.Galectin-3 expression was significantly more common in malignant FPTLs (FVPTC) when value compared to NIFTP and benign FPTLs (HNs and FA)(P value 0.000). A summary of these findings is shown in Tables 3 and 4.

CK19 protein immunoreactivity was not observed in the normal follicular epithelium. CK19 protein staining was detected predominantly in the cytoplasm in carcinomas. CK19 protein expression was detected in 20/51 cases of benign lesions (44.7% HNs, and 0% FA), focally in 3/12 (25%) in NIFTP, and in 14/20 in carcinomas (64.7% FVPTC and 100% FC). CK19 expression was absent in 31/51 cases of benign FPTLs (55.3% of HNs and 76.9% of FA), in 9/12 (75 %) NIFTP, in 6/17 of FVPTC, and in none of the FC. CK19 protein expression was significantly more common in malignant FPTLs (FVPTC and FC) when compared to NIFTP and benign FPTLs (HNs, and FA, P value 0.02). The expression in carcinomas (cytoplasmic ± membranous) was more diffuse and had strong staining intensity. In benign FPTLs, CK19 staining was patchy and had weak staining intensity. A summary of these findings is shown in Tables 3 and 4. 

CD56 protein immunoreactivity was observed in the normal follicular epithelium.CD56 protein reactivity was observed predominantly in the cytoplasm in the benign FPTLs. CD56 protein expression was seen 32/51 of benign lesions (73.7% HNs, and 30.8% FA), in 2/12 (16.7 %) NIFTP and in 12/20 carcinomas (52.9%FVPTC and 100%FC).CD56 was absent in 19/51 cases of benign lesions (26.3% of HNs and 69.2% of FA), in 10/12 (83.3%) NIFTP, and in 8/17 (47.1%) FVPC and in 0/3 (0%) FC.CD56 protein expression was significantly more common in benign FPTLs (HNs, and FA) as compared to malignant FPTLs (FVPTC and FC) and NIFTP (P value 0.003).A summary of these findings is shown in Tables 3 and 4. 

The sensitivity, specificity, and accuracy rate of the immunostains: Table 5 demonstrates the sensitivity, specificity, and accuracy rate of HBME-1, Galectin-3, CK19, and CD56 to separate benign FPTLs from malignant ones (FVPTC and FC). The sensitivity of CK19 for diagnosis of carcinomas (FVPTC and FC) 70% and the specificity is 63.5%. The sensitivity of galectin-3 for diagnosis of carcinomas (FVPTC and FC) is 75% and the specificity is 90.5%. The sensitivity of CD56 for diagnosis of carcinomas (FVPTC and FC) is 60% and the specificity is 46%. HBME-1 and galectin-3 had the highest sensitivity (75%) but galectin-3 was the most specific (90.5%) and was the most accurate immunohistochemical marker (86.7%). Therefore HBME-1 and Galectin-3 were the most sensitive markers for the diagnosis of malignant FPTLs (75%), whereas Galectin-3 was the most specific one (90.3%). CK 19 has good sensitivity (70%) for the diagnosis of carcinomas. CD56 had the least sensitivity or specificity for the diagnosis of carcinomas. A summary of these findings is shown in Tables 5 and 6. The co-expression of HBME-1 and CK19 has a sensitivity of 45 % and specificity of 55.6% in the diagnosis of malignant FPTLs (FVPTC and FC). The co-expression of HBME-1 and Galectin-3 has a sensitivity of 60 % and specificity of 73% in the diagnosis of malignant FPTLs (FVPTC and FC). The co-expression of Galectin-3 and CK19 has a sensitivity of 45 % and specificity of 63.5 % in the diagnosis of malignant FPTLs (FVPTC and FC). The simultaneous use of HBME-1, Galectin-3, CK19, and CD56 markers has a sensitivity of 15% and specificity of 22.2% in the diagnosis of malignant FPTLs (FVPTC and FC).

## Discussion

The FPTLs represent a challenging issue in thyroid pathology (Modi and Daveshwar, 2018). Histologically, they have some overlapping morphological features and therefore represent a diagnostic dilemma for the practicing pathologists. For instance, the histological distinction between benign (HNs and FA) and malignant FPTLs (FVPTC and FC) is sometimes difficult, therefore additional diagnostic reliable immunostains are needed. To date, there is an increasing number of markers in the process of continuous evaluation for their diagnostic utility. Although there are some previous reports about the diagnostic utility of HME-1, Galectin-3, CK19, and CD56 immunostains in the separation of PTC from papillary hyperplasia and HNs, we still have limited knowledge about the expression patterns of these proteins in a wide spectrum of FPTLs. Moreover, a combination of these markers was not previously examined in NIFTP. We carried this study to address this issue. Here we investigated the diagnostic applicability of four immune markers in the separation of 83 benign and malignant FPTLs. 

Our study demonstrated the following: (i) all the markers are expressed in both benign and malignant FPTLs, indicating that to date there is no reliable immunostain that can stand alone and resolve the issue, (ii) statistically significantly more frequent expression of HBME-1, CK19, Galectin-3 proteins in carcinomas as compared to benign FPTLs (p = <0.01), (iii) HBME-1 and Galectin-3 were the most sensitive markers for the diagnosis of malignant FPTLs (75%), and (iv) Galectin-3 was the most specific one (90.3%). Moreover, we observed that although some of these markers are promising to separate benign from malignant FPTLs, none stand alone reliable for differential diagnosis. Each marker has its limitations because of significant expression in benign and malignant thyroid nodules. Therefore, novel immunohistochemical markers or combinations of the old markers are required for an accurate diagnosis.

HBME-1 protein expression in FPTLs: HBME-1 monoclonal antibody can react with yet uncharacterized antigen in the microvilli of mesothelial cells. In concordance with previous reports (Barut et al., 2010; Erdogan-Durmus et al., 2016; Wang et al., 2019), we found expression of HBME-1 protein in both malignant (FVPTC and FC, diffuse and intensively strong staining pattern) and benign FPTLs (HNs and FA, focal, weak staining pattern). Barut et al examined the utility of HBME-1, galectin-3, and CK19 in the distinction of benign and malignant thyroid lesions (Barut et al., 2010). The expressions of these markers were examined in formalin-fixed, paraffin-embedded tissues of 458 FPTLs. In carcinomas, all of these markers (including HBME-1 protein) were diffusely expressed. Although focal galectin-3, HBME-1, and cytokeratin-19 expression were encountered in benign FPTLs, diffuse positive reactions for these three markers were more characteristic of carcinomas (Barut et al., 2010). The presence of HBME-1, CK19, Galectin protein expression in both benign and malignant FPTLs may be reasoned to the fact that all of these lesions have the same pluripotent stem cells origins, which proved that the association between conditions is antibody specific and may have an oncogenic role(Kim et al., 2009; Azizi and Malchoff, 2011)

Galectin-3 protein expression in FPTLs: Galectin-3 is a member of the β-galactoside-binding mammalian family of lectins that serves functions in metastasis, angiogenesis, proliferation, and apoptosis of multiple tumor types, including thyroid carcinoma (Huang et al., 2018). In our series, we found a diffuse strong Galectin-3 protein expression in FVPTC. In the benign FPTLs, the expression was infrequent, focal, and weak. These findings concur with previous studies (Rosahl et al., 2000; Jakubiak-Wielganowicz et al., 2003; Takano et al., 2005; Chen et al., 2006) and indicate that Galectin-3 is a very useful marker for the diagnosis of carcinoma, i.e. as the most specific marker for FVPTC. Moreover, Galectin-3 may be the stand-alone immunostain for the distinction between benign and malignant FPTLs (Wiseman et al., 2008). In the FPTLs, Galectin-3 may represent a phenotypic marker and an oncogenic element. Galectin 3 may be involved in the development of malignant FPTLs by making cross-links with cell membrane glycoproteins. The resulting network is involved in cellular signaling, apoptosis, cell motility, and it is involved in thyroid carcinoma progression (Xu et al., 1995; Liu and Rabinovich, 2005).

CK19 protein expression in FPTLs: CK19 is an intermediate filament protein with a molecular weight of around 40 kDa. It is the smallest member of the cytokeratin family, responsible for the structural integrity of epithelial cells. It is absent from the healthy thyroid follicular cells (Kakudo et al., 2012). Several studies reported that CK19 expression is intensely and diffusely expressed in PTC and absent or faintly expressed in the benign thyroid lesions which makes this marker useful in the diagnosis of separation of these lesions (Arcolia et al., 2017; Huang et al., 2018). In agreement with the previous reports (Arcolia et al., 2017; Huang et al., 2018), we observed diffuse strong CK19 protein expression in FVPTC and FC. Although CK19 protein expression was also seen in some HNs, FA, and NIFTP, the staining was weak, foal and patchy (Barroeta et al., 2006; Zhu et al., 2010). Therefore the separation of malignant FPTLs from the benign ones may be based on the distribution and intensity of the staining of CK19, i.e. the presence of a diffuse intensively strong cytoplasmic ± membranous accentuation should raise the possibility of malignancy even in absence of the morphological features of PTC (Dunderovic et al., 2015). Cytokeratin-19 alone and in combinations with other markers such as HBME-1 and Galectin-3 were more sensitive for an accurate diagnosis of PTC and FC than the other combinations (Barut et al., 2010). In the FPTLs, whether CK19 represents a phenotypic marker or an oncogenic element is still largely unknown. Several pathways regulating CK19 protein expression may play roles in carcinogenesis. They include some transcription factors, carcinogenic growth factors and corresponding receptors, MAKP/JNK and MEK-ERK1/2 pathways, and noncoding RNAs (Zhuo et al., 2020).

CD56 protein expression in FPTLs: CD56 is a neural adhesion molecule that associates with the fibroblast growth factor receptor and stimulates the tyrosine kinase activity of receptors to induce neurite outgrowth. It is found in normal thyroid follicular cells and is related to their differentiation. It is highly expressed in the normal and non-neoplastic and benign thyroid tissues (Park et al., 2009; Abd El Atti and Shash, 2012; Abdou et al., 2018). In this study, we found CD56 positivity in more than half of thyroid carcinomas. It was negative in most of FAs, and most of NIFTP nodules. The downregulation of CD56 is linked to tumor progression. The lack of CD56 protein expression in the malignant (FVPTC and FC) in our series concurs with previous results (Erdogan-Durmus et al., 2016; Harb, 2017; Pyo et al., 2018). Abd El Atti examined the role of CD56 in discriminating the follicular variant of PTC from other solitary follicular patterned nodules. The immunohistochemical expression of CD56 and claudin-1 was evaluated in 86 samples of thyroid lesions together with 10 samples of normal thyroid tissue. There was a significantly different expression of CD56 in the follicular variant of PTC versus other solitary follicular patterned nodules (Abd El Atti and Shash, 2012). Taken together, it can be deduced that CD56 had low sensitivity and specificity for the diagnosis of thyroid carcinoma (Dunđerović et al., 2015). Although CD56 is a good negative marker for diagnosing conventional variant of PTC (Park et al., 2009), it is of less value in follicular patterned malignant nodules.

The analysis of the expression pattern of HBME-1, Galectin-3, CK19, and CD56 in NIFTP: NIFTP (also previously known as encapsulated non-invasive FVPTC) is a new contribution of this current study to the current field of diagnostic markers in FPTLs. The expression patterns of these markers in NIFTP were more similar to those of benign FPTLs than to thyroid carcinomas, attesting to their proposed premalignant nature. In agreement with Cho et al, we found that most of the cases of NIFTP, were positive for HBME-1, while only focal positivity was observed for CK 19, galectin-3, and CD56(Cho et al., 2018). NIFTP are considered as borderline lesions that occupy a middle ground between FA and invasive FVPTC or minimally invasive FC, i.e. ‘pre-malignant’ follicular proliferation with RAS mutations (Rosario and Mourao, 2019). 

Sensitivity and specificity of HBME-1 and Galectin-3 in FPTLs: In this study, CK19 was expressed in malignant FPTLs with high sensitivity, specificity, and negative predictive value. These findings are in agreement with other studies (Dunđerović et al., 2015; Tastekin et al., 2019). Moreover, in concordance with other studies, our work revealed that HBME-1 (Saleh et al., 2010; Palo and Biligi, 2017; Zargari and Mokhtari, 2019) and Galectin-3 (Saleh et al., 2010; Muzafar et al., 2017) had the highest sensitivity for the diagnosis of thyroid carcinoma. Javalgy et al., (2019) and AbuSinna et al., ( 2018) reported higher sensitivity and specificity for HBME-1 than Galectin-3 while Dunderovic et al., (2015) found higher sensitivity for Galectin-3 and higher specificity for HBME-1. The discordance between our work and other studies is reasoned to the inclusion of the conventional variant of PTC in their work. Galectin-3 in the current study had the highest positive predictive value, negative predictive value, accuracy, and was more specific for the diagnosis of thyroid carcinoma. Taken as a whole, it can be deduced that the combined use of galectin-3, HBME-1and CK19 improves the sensitivity and diagnostic accuracy of thyroid nodules(Abu-Sinna et al., 2018). 

To conclude, FPTLs remain a diagnostic dilemma for pathologists and clinicians. The discrepancy regarding their diagnostic utility among the different studies is reasoned to the use of different cut off values, lack of consensus about the pattern of staining of these proteins (cytoplasmic ± membranous). HBME-1, Galectin-3, and CK19 are frequently expressed in malignant FPTLs. However, they are also inconsistently expressed in the benign FPTLs. Therefore, it is prudent to use all of the four markers in combination can help differentiate carcinomas from HNs and FA. Moreover, evaluation of the routine H/E stained sections will continue to be the golden standard for the diagnosis of FPTLs until future studies can find more reliable markers.

**Table 1:** The Antibodies with Their Clones, Antigen Retrieval Methods, and Staining Patterns

	Anti-HBME-1	Anti-cytokeratin 19	Anti-Galectin-3	Anti-CD56
Antigen Retrieval	Citrate buffer	EDTA	EDTA	EDTA
Dilution	1:50	0.111111111	1:25	1:25
Time of incubation	1 hour	1 hour	Overnight	Overnight
Positive control	Mesothelioma	Ureter	PTC	Small cell carcinoma
Clone	HBME-1	A53-B/A2.26	9C4	123C3
Catalog no	61-0127-2	61-0163-2	61-0133-2	61-00662

**Table 2:** The Clinicopathologic Features of Follicular Ptterned Thyroid Lesions

Aspects	Benign FPTLs			Malignant FPTLs	
	HN	FA	NIFTP	FVPTC	FC
Number of cases	38	13	12	17	3
Mean of age (years)	33.1	40	36.7	41.5	45
Sex (M:F)	01:02.8	01:02.3	1:02	01:02.4	1:02
Clinical presentation					
Diffuse goiter	15	0	0	5	0
Solitary nodule	8	13	12	7	3
Multiple nodules	15	0	0	5	0
Surgical management					
Subtotal thyroidectomy	30	3	4	11	3
Lobectomy	8	10	8	6	0
Gross features					
Non-encapsulated	28	0	0	7	0
Thin capsule	10	13	12	10	0
Thick capsule	0	0	0	0	3
Capsular invasion	0	0	0	0	3
Lymphovascular invasion	0	0	0	10	3
Metastatic deposits in the regional lymph nodes	0	0	0	7	0
Non-nodal metastatic deposits	0	0	0	1	1

**Table 3:** Frequency of Expression of HBME-1, Galectin-3, CK19, and CD56 in the benign and Malignant Follicular Patterned Thyroid Lesions and Non-Invasive Follicular Neoplasm with Papillary-Like Nuclear Features

	No. of cases	HBME-1	CK19	Galectin-3	CD56
Benign	51	10	20	3	32
		-19.60%	-39.20%	-5.90%	-62.70%
NIFTP	12	7	3	3	2
		-58.30%	-25%	-25%	-16.70%
Malignant	20	15	14	15	12
		-75%	-70%	-75%	-60%
P value		0.000*	0.02*	0.000*	0.014

The benign FPTLs include HNs and FA. The malignant FPTLs include FVPTC and FC; Differences in the frequency of expression of HBME-1 between malignant FPTLs (FVPTC and FC) versus benign FPTLs (HNs and FA) (HBME-1): χ2= 19.32, p-value= 0.000; Differences in the frequency of expression of CK19 between malignant FPTLs (FVPTC and FC) versus benign FPTLs (HNs and FA) (HME-1): χ2= 5.456; , p-value= 0.02 Differences in the frequency of expression of Galectin-3 between malignant FPTLs (FVPTC and FC) versus benign FPTLs (HNs and FA) (HME-1): χ2= 36.265, p-value= 0.000; Differences in the frequency of expression of CD56 between malignant FPTLs (FVPTC and FC) versus benign FPTLs (HNs and FA) (HME-1): χ2= 0.046, p-value= 0.83

**Table 4:** Expression of HBME-1, Galectin-3, CK19, and CD56 in the Hyperplastic Nodules, Follicular Adenomas, Follicular Variant of Papillary Thyroid Carcinoma, Non-Invasive Follicular Neoplasm with Papillary-Like Nuclear Features, and Follicular Carcinoma

Diagnosis		HBME-1	CK19	Galectin-3	CD56
HNs	Positive	0 0%	7 -18.40%	0 0%	15 -39.50%
	Focally positive	6 -15.80%	10 -26.30%	3 -7.90%	13 -34.20%
	Negative	32 -84.20%	21 -55.30%	35 -92.10%	10 -26.30%
FA	Positive	1 -7.70%	0 0%	0 0%	0 0%
	Focally positive	3 -23.10%	3 -23.10%	0 0%	4 -30.80%
	Negative	9 -69.20%	10 -76.90%	13 -100%	9 -69.20%
NIFTP	Positive	7 -58.30%	0 0%	0 0%	0 0%
	Focally positive	0 0%	3 -25%	3 -25%	2 -16.70%
	Negative	5 -41.70%	9 -75%	9 -75%	10 -83.30%
FVPC	Positive	12 -70.60%	6 -35.30%	9 -52.90%	3 -17.60%
	Focally positive	0 0%	5 -29.40%	6 -35.30%	6 -35.30%
	Negative	5 -29.40%	6 -35.30%	2 -11.80%	8 -47.10%
FC	Positive	3 -100%	3 -100%	0 0%	0 0%
	Focally positive	0 0%	0 0%	0 0%	3 -100%
	Negative	0 0%	0 0%	3 -100%	0 0%
P value*		0	0.003	0	0.001

HNs: hyperplastic nodule, FA: Follicular adenoma, NIFTP: Non invasive follicular tumor with papillary like nuclear features, FVPC: Follicular variant of papillary carcinoma, FC: Follicular carcinoma; Differences in the frequency of expression of HBME-1 between FVPTC versus HNs: χ2= 16.02, pvalue= 0.000;Differences in the frequency of expression of HBME-1between FVPTC versus FA: χ2= 4.693, pvalue= 0.03; Differences in the frequency of expression of HBME-1between FC versus FA: χ2= 4.747, p-value= 0.029; Differences in the frequency of expression of HBME-1between FVPTC versus NIFTP: χ2= 0.468, p-value= 0.494; Differences in the frequency of expression of Galectin-3between FVPTC versus HNs: χ2= 34.43, p-value= 0.000; Differences in the frequency of expression of Galectin-3between FVPTC versus FA: χ2= 22.94, p-value= 0.000; Differences in the frequency of expression of Galectin-3between FVPTC versus NIFTP: χ2= 11.948, p-value= 0.001; Differences in the frequency of expression of CK19between FVPTC versus HNs: χ2= 1.874, p-value= 0.171; Differences in the frequency of expression of CK19between FVPTC versus FA (CK19): χ2= 5.129, p-value= 0.024; Differences in the frequency of expression of CK19 between FC versus FA: χ2= 6.154, p-value= 0.013; Differences in the frequency of expression of CK19 between FVPTC versus NIFTP: χ2= 4.441, p-value= 0.035; Differences in the frequency of expression of CD56 between FVPTC versus HNs: χ2= 2.295, p-value= 0.13; Differences in the frequency of expression of CD56 between FVPTC versus FA: χ2= 1.475, p-value= 0.23; Differences in the frequency of expression of CD56 between FC versus FA: χ2= 4.747, p-value= 0.029; Differences in the frequency of expression of CD56 between FVPTC versus NIFTP: χ2= 3.932, p-value= 0.047

**Table 5:** The Sensitivity and Specificity, Predictive Values and Likelihood Ratios of the HBME-1, Galectin-3, CK19 and CD56 in the Differentiation of Malignant versus benign Follicular Patterned Thyroid Lesions

	SEN	SPE	PPV	NPV	PLR	NLR	ACC
HBME-1	75%	73%	46.90%	90.20%	2.78	0.34	73.50%
CK19	70%	63.50%	37.85%	87%	1.92	0.47	65.10%
Galectin-3	75%	90.50%	71.40%	91.90%	7.89	0.28	86.70%
CD56	60%	46%	26.10%	78.40%	1.11	0.87	49.40%

SEN, sensitivity; SPE, specificity; PPV, positive predictive value; NPV, negative predictive value; PLR, positive ; likelihood ratio, NLR, negative likelihood ratio; ACC, accuracy.

**Table 6:** The Sensitivity and Specificity of the HBME-1, Galectin-3, CK19 and CD56 in the Differentiation of Malignant versus benign Follicular Patterned Thyroid Lesions

Marker		M/B	FVPTC/HNs	FVPTC/ FA	FVPTC/ NIFTP	FC/HNs	FC/FA	FC/NIFTP
HBME-1	Sensitivity	75%	70.60%	70.60%	70.60%	33.30%	100%	100%
	Specificity	80.40%	84.20%	69.20%	41.70%	84.20%	69.20%	41.70%
Galectin-3	Sensitivity	75%	88.20%	88.20%	88.20%	0%	100%	0%
	Specificity	94.10%	92.10%	100%	75%	92.10%	100%	75%
CK19	Sensitivity	70%	64.70%	64.70%	64.70%	100%	100%	100%
	Specificity	60.80%	55.30%	76.90%	75%	55.30%	76.90%	75%
CD56	Sensitivity	60%	52.90%	52.95	52.90%	100%	100%	100%
	Specificity	37.30%	26.30%	69.20%	83.30%	26.30%	69.20%	83.30%

Malignant FPTLs (M) versus benign FPTLs (B).

## Author Contribution Statement

Dr. Dalia A Elsers; Conceptualization, data curation, funding acquisition, project administration, methodology , investigation, resources ,supervision, validation, visualization , review and editing. Prof Dr. Mahmoud R A Hussein; Conceptulaization, data curation, funding acquisition, project administration, methodology, investigation, supervision, visualization ,validation, original draft , review and editing. Dr. Mohammed Hasssan Osman; Conceptualization, data curation, funding acquisition, methodology, review and editing. Dr. Ghada A Mohamed; Validation, Methodology and Review
